# Riddles of Lost City: Chemotrophic Prokaryotes Drives Carbon, Sulfur, and Nitrogen Cycling at an Extinct Cold Seep, South China Sea

**DOI:** 10.1128/spectrum.03338-22

**Published:** 2022-12-13

**Authors:** Yu Chen, Yuanjiao Lyu, Jian Zhang, Qiqi Li, Lina Lyu, Yingli Zhou, Jie Kong, Xinyang Zeng, Si Zhang, Jie Li

**Affiliations:** a Southern Marine Science and Engineering Guangdong Laboratory (Guangzhou), Guangzhou, Guangdong, People’s Republic of China; b CAS Key Laboratory of Tropical Marine Bio-resources and Ecology, South China Sea Institute of Oceanology, Chinese Academy of Sciences, Guangzhou, Guangdong, People’s Republic of China; Nanjing Institute of Geography and Limnology, Chinese Academy of Sciences

**Keywords:** cold seep, extinct phase, chemotrophic, prokaryotes, geochemical cycling

## Abstract

Deep-sea cold seeps are one of the most productive ecosystems that sustained by hydrocarbons carried by the fluid. Once the seep fluid ceases, the thriving autotrophic communities die out, terming as the extinct seep. But heterotrophic fauna can still survive even for thousands of years. The critical role of prokaryotes in active seeps are well defined, but their functions in extinct seeps are poorly understood to date. Here, we clarified the diversity, taxonomic specificity, interspecies correlation, and metabolic profiles of sediment prokaryotes at an extinct seep site of Haima cold seep, South China Sea. Alpha diversity of archaea significantly increased, while that of bacteria remained unchanged in extinct seep compared to active seep. However, archaea composition did not differ significantly at extinct seep from active or nonseep sites based on weighted-unifrac dissimilarity, while bacteria composition exhibited significant difference. Distribution of archaea and bacteria showed clear specificity to extinct seeps, indicating the unique life strategies here. Prokaryotes might live chemolithoautotrophically on cycling of inorganic carbon, sulfur, and nitrogen, or chemoorganotrophically on recycling of hydrocarbons. Notably, many of the extinct seep specific species and networked keystone lineages are classified as Proteobacteria. Regarding the functional diversity and metabolic flexibility of this clade, Proteobacteria is supposed to integrate the geochemical cycles and play a critical role in energy and resource supplement for microbiome in extinct seep. Collectively, our findings shed lights on the microbial ecology and functional diversity in extinct seeps, providing new understanding of biogeochemical cycling after fluid cessation.

**IMPORTANCE** This research paper uncovered the potential mechanisms for microbiota mediated geochemical cycling in extinct cold seep, advancing our understanding in deep sea microbiology ecology.

## INTRODUCTION

Cold seeps are first discovered in 1983 and considered one of the most productive deep-sea ecosystems (1). The cold seep biota consumes methane for carbon and energy sources, and terms ‘the benthic filter for methane’, which influences the amount of methane emitted from the sea floor (2–4). Methane-rich fluid emitted from the sedimentary subsurface sustains the chemotrophic organisms and fundamentally affects the transition of fauna and microbial communities (5). Fauna aggregates consisted of different species, such as Vesicomyidae clams, mussels, and tubeworms, occur in turn and are highly constrained in a specific sequence, signing the succession of cold seep fauna (5). Previous studies at seeps in the Gulf of Mexico and Hikurangi Margin suggested the role of environmental variables, such as methane and sulfide, in fauna community succession (5–7). But once the fluid ceased, the chemoautotrophic fauna are going to die out, symbolized by widespread perishment of organisms, as well as the appearance of carbonate rocks or platform (5), like the lost city. Methane outage results in failures of chemosynthetic organisms, and thus leading to dramatic declines of biomass and biodiversity (8). However, regardless of the diminishment of autotrophic faunas, heterotrophic fauna aggregates can survive on the extinct seep after the fluid ceased, even for thousands of years (9), emphasizing the significance and particularity of extinct seep ecosystem. Sea anemones, cold-water corals, non-methanotrophic sponges, and Crustacean species are ubiquitous colonizers of extinct cold seep, though some of them are also found in active seeps (5, 8). Tubeworms are supposed to be the last chemoautotroph-depending fauna aggregates in extinct seeps (5, 10). But there is an obvious gap, more than 1,000 years, between the dying out of tubeworm aggregates and colonization of corals (9). These clues indeed indicate a sequential succession of biota after seepage ceased, but little is known about the ecological roles of faunas and microbiome in extinct seeps.

Faunas inhabiting in extinct seeps are originally considered to live on the remains of mussels or tubeworms (5, 11, 12). However, the following studies have proved the involvement of chemoautotrophic prokaryotes in nutrition acquisition of these heterotrophic organisms. Sponge associated microbiome is comprised of Gammaproteobacteria that carrying the *pmoA* gene responsible for aerobic methane oxidation (13). A recent study in an extinct seep at Central Arctic seamounts further reveals that the autotrophic symbiotic prokaryotes contribute significantly to carbon assimilation of the giant bacteriosponges (14), suggesting the importance of chemosynthetic prokaryotic consortia in extinct seeps. Additionally, isotopic clues from an inactive hydrothermal vent also support the fact that the food web is dominantly relying on production of chemoautotrophic microbiome (15). These findings implies that prokaryotes may play a role in nutrient sustainment for faunas at extinct seeps. Moreover, many studies have revealed that authigenic carbonate extensively develops after dying out of the fauna communities, which is supposed to be driven by microbial metabolism (12, 16–18). In ancient methane seep reefs, for example the Jiulong methane reef at South Chia Sea (SCS) and the Crimea peninsula at Black Sea, anaerobic methanotrophs (ANME) affiliated with Euryarchaea and sulfate reducing bacteria (SRB) belonging to Deltaproteobacteria are identified with high abundance, who are actively mediating anaerobic oxidation of methane (AOM) coupled sulfate reduction (SR) (16, 18). Other to these taxa, Alphaproteobacteria, Betaproteobacteria, Gammaproteobacteria, Planctomycetes, Chloroflexi, and Bacteroidetes are also abundant in extinct seeps (13, 19), suggesting a mix nutrition approach pattern for extinct seep prokaryotes. However, we cannot get more information regarding extinct seep prokaryotes from the current studies. What roles do they play in food web and biogeochemical cycling in extinct seeps, and how do they function together? These questions are to be addressed.

Similar to cold seeps, hydrothermal vents are also fluid-sustaining ecosystems. A pronounced shift in structure of microbial communities is found upon fluid stops in seeps and vents (13, 19, 20). Previous studies have found that sulfide deposits delivered by the vent fluid remain abundantly colonized by chemoautotrophic prokaryotic consortia even after hydrothermal venting has ceased (21). This finding inspired us that the “fluid deposit” refractory hydrocarbons, inclusive of polycyclic aromatic hydrocarbons (PAHs), long-chain hydrocarbons, etc. are probable source for prokaryotes in extinct seeps (22). Thus, we may infer the role of prokaryotes in extinct seeps from the inactive hydrothermal vents. For instance, Gammaproteobacteria species are major primary productors in inactive vents globally, utilizing sulfur, iron and hydrogen as electron donors coupled with oxygen and nitrate respiration (20, 23, 24). Nitrospirae is identified as an indicator for early ceasing vent, mediating sulfide minerals oxidation coupled oxygen, nitrate, and sulfate reduction (20). Bacteroidota and Patescibacteria are potentially responsible for fermentative recycling of organic carbon (24). Thus, geochemical cycling is driven by prokaryotes in inactive vents, partially supporting the energy demand of community with chemosynthesis. Heterotrophic species play a key role in recycling of organic compounds, as an essential supplement for primary production. In addition, the metabolic pathways for microbial carbon, nitrogen, and sulfur cycles were well defined in marine sediment to date. Key genes may infer the presence of a given metabolic pathway, such as the *dch* gene in benzoyl-CoA pathway for aromatic hydrocarbon degradation (25), the *dsrA/B* genes in dissimilatory sulfate reduction pathway (26), and the *nirS/K* genes in denitrification pathway mediating NO production (27), making it possible to profile the geochemical functions of a given prokaryotic community.

**FIG 1:**
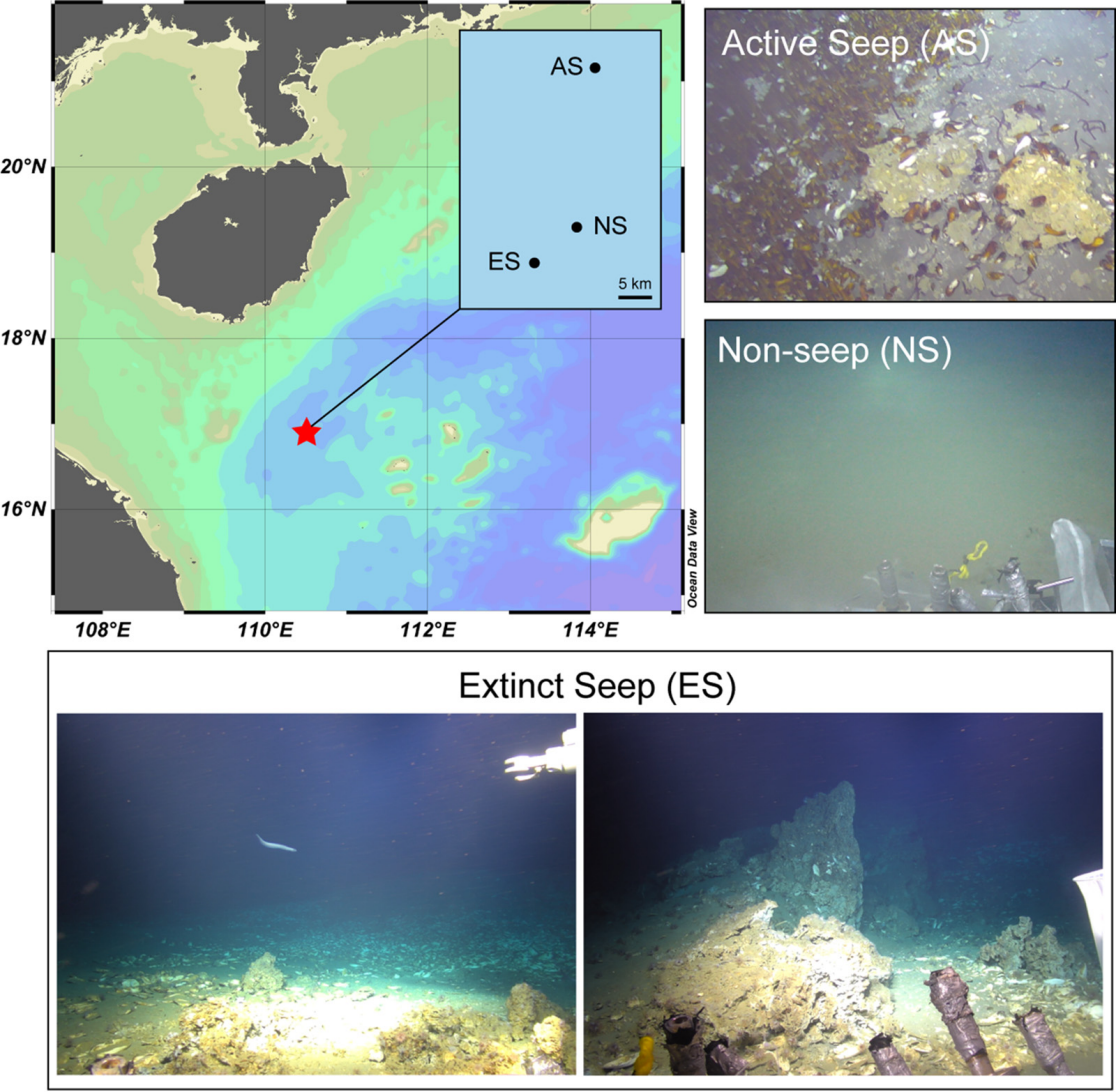
Location and seafloor images of different sites of Haima cold seep, SCS. The active seep site (AS) inhabits thriving fauna aggregates. The nonseep site (NS) is a muddy seafloor without faunas. In the extinct seep site, shells of dead mussels and clams, heterotrophic sea anemone are found. Large autogenic autogenetic carbonate rocks are considered the footprint of cold seep. The photos are taken by the Haima Camara carried by a remote operational vehicle.

Here, in this study, we are reporting the findings on prokaryotic diversity and functions at an extinct seep site in Haima cold seep, SCS, identified by the widespread perishment of bivalves and large authigenic carbonate rocks (Fig. 1). Changes in community diversity and composition, potential interspecies correlation, and metabolic functions are evaluated via meta-omics approaches. Through this work, we are aiming at profiling both the structure and the function of prokaryotic community at extinct seep site, advancing our understanding of integration of biogeochemical cycling and prokaryotic community transition after cold seep fluid ceased.

## RESULTS

### Composition and diversity of archaea and bacteria at different sites.

**FIG 2:**
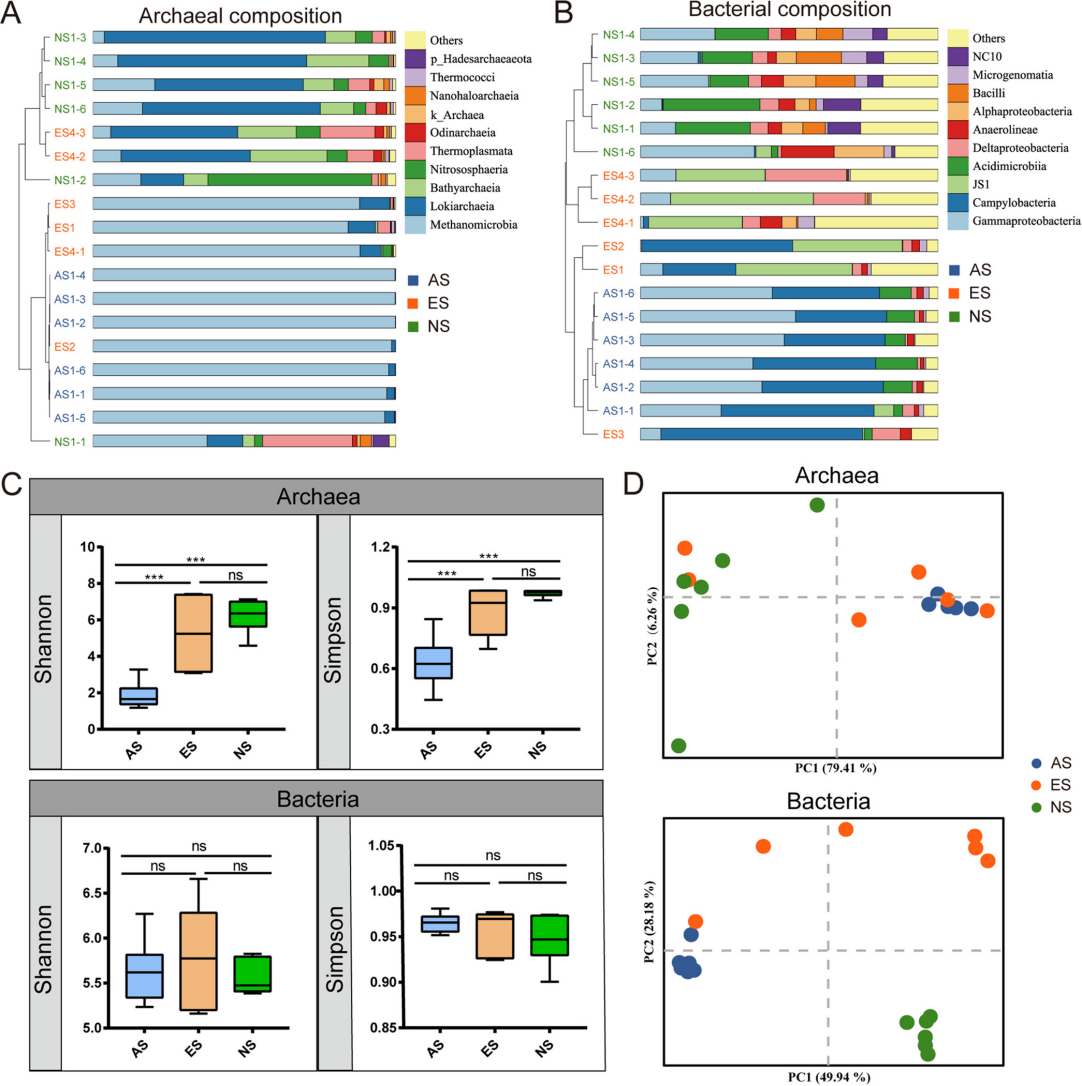
Composition and diversity of archaea and bacteria. (A, B) Relative abundance of dominant archaea and bacteria orders at AS, ES, and NS sites. The samples were clustered via UPGMA based on weighted unifrac distance. The color of sample name represented the sampling site. AS, blue; ES, orange; NS, green. (C) Variation of Shannon and Simpson indexes for archaea and bacteria communities at different sites. (one-way ANOVA adjusted with Tukey, ***, *P* < 0.001; ns, not significant). (D) Principle coordinate analysis (PCoA) for archaea and bacteria communities based on weighted unifrac distance.

We investigated the composition and diversity of prokaryotes of an active seep site (AS), an extinct seep site (ES), a nonseep site (NS) in Haima cold seep, SCS (Fig. 1, Table S1, Supplementary Movie). Methane concentration of bottom water was >9.9 μM, 0.11 μM, and 0 μM at AS, ES, and NS site, respectively. A total of 653,729 and 612,972 full-length 16S rRNA gene sequences were retrieved and rarefied by the minimum sample size for archaea (5714) and bacteria (7847), respectively. The rarefaction curve analysis suggested the sufficiency of sample size for community diversity evaluation (Fig. S1). For the archaea community, Methanomicrobia (96.76 to 99.84%) was predominant in archaea community of AS samples. At NS site, Methanomicrobia (3.71 to 37.66%), Lokiarchaeia (11.81 to 73.06%), Bathyarchaeia (3.85 to 20.52%), Nitrososphaeria (2.71 to 54.07%), and Thermoplasmata (4.06 to 29.42%) dominantly comprised the archaea community. Composition of archaea was divergent between different samples of ES site. Methanomicrobia (84.29 to 98.63%) was predominant in ES1, ES2, ES3, and ES4-1, similar to the AS samples. While archaea composition of ES4-2 and ES4-3 was similar to the NS samples based on weighted unifrac dissimilarity UPGMA cluster analysis (Fig. 2A). For the bacteria community, Gammaproteobacteria (27.09 to 52.09%) and Campylobacteria (30.66 to 51.41%) were dominant in the AS site. The JS-1 clade (29.96 to 48.05%) was prevalent at ES site, while Campylobacteria (24.51 to 67.83% in ES1, ES2, and ES3) and Deltaproteobacteria (5.98 to 27.32% in ES4 samples) were also abundant. Gammaproteobacteria (7.17 to 38.25%), Acidimicrobiia (2.19 to 32.56%), Deltaproteobacteria (1.18 to 6.30%), Anaerolineae (2.89 to 17.71%), Bacilli (2.21 to 15.21%), Microgenomatia (0.88 to 9.96%), and NC10 clade (1.09 to 12.55%) dominantly comprised the bacteria community of NS site (Fig. 2B). Shannon and Simpson indexes of archaea community showed significant increase at ES and NS sites compared to the AS site (Shannon, One-way ANOVA, *P* < 0.001; Simpson, One-way ANOVA, *P* < 0.001). While that of bacteria community did not change among the three sites (Shannon, One-way ANOVA, *P* = 0.61; Simpson, One-way ANOVA, *P* = 0.37; Fig. 2C). Weighted-unifrac dissimilarity based principal coordinate analysis (PCoA) showed that archaea community of AS and NS sites exhibited non-overlap pattern, while the ES samples clustered with both sites. The distinct archaea communities at AS and NS sites were confirmed by analysis of similarity (ANOSIM, *R* = 0.749, *P* = 0.008) and permutational multivariate analysis of variance (PERMANOVA, R^2^ = 0.956, *P* = 0.008). However, significant difference was not found between the archaea communities of ES site and AS or NS site by PERMANOVA (AS versus ES, R^2^ = 0.956, *P* = 0.09; NS versus ES, R^2^ = 0.956, *P* = 0.09, Table 1). Bacteria community showed clear separation between all sites (Fig. 2D), and their dissimilarity was further inferred by ANOSIM (*R* = 0.813, *P* < 0.001, Table 1) and PERMANOVA analyses (R^2^ = 0.636, *P* < 0.001, Table 1).

**TABLE 1:** ANOSIM and PERMERNOVA analysis for archaea and bacteria communities^*a*^

Site	Archaea				Bacteria			
	ANOSIM		PERMERNOVA		ANOSIM		PERMERNOVA	
	R	*P*	R^2^	*P*	R	*P*	R^2^	*P*
AS/NS	0.956	**0.008**	0.749	**0.008**	0.767	**0.008**	1	**0.008**
AS/ES	0.37	**0.008**	0.257	0.09	0.656	**0.008**	0.592	**0.008**
ES/NS	0.304	**0.032**	0.271	0.054	0.798	**0.008**	0.446	**0.008**

a Bold indicated *P* < 0.05.

### Distribution specificity of archaea and bacteria species at different sites.

**FIG 3:**
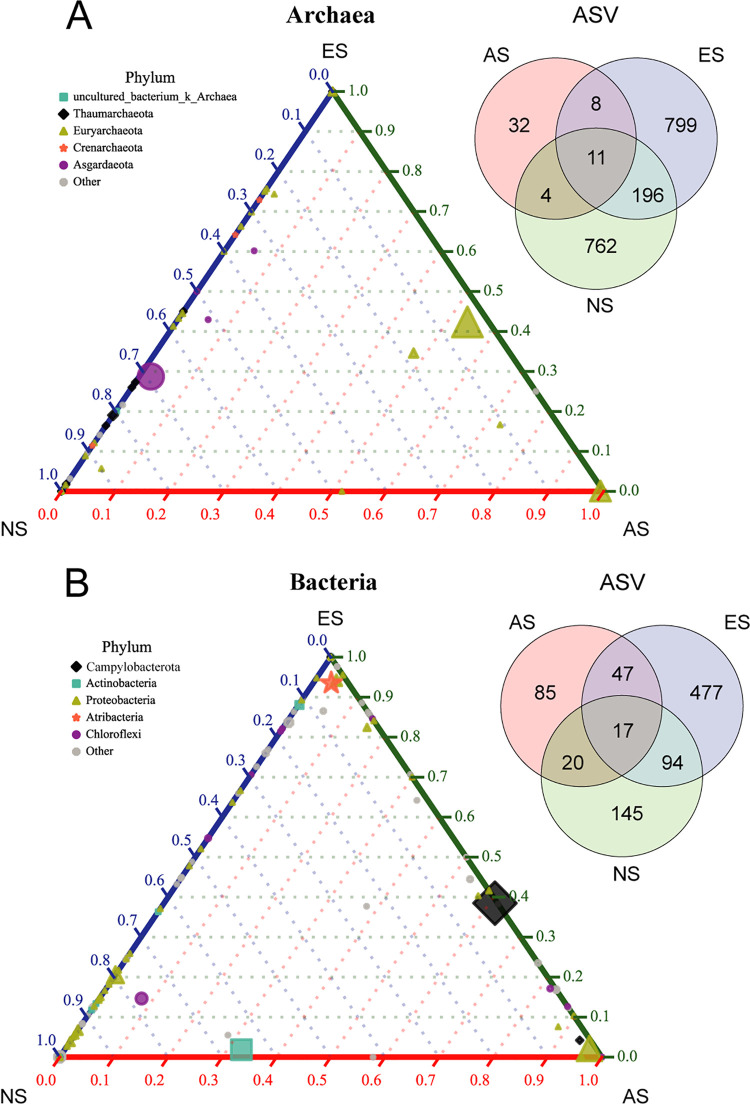
Distribution specificity of archaea and bacteria. Archaea (A) and bacteria (B) distribution were illustrated with ternary plotting on species level. Size of the plots indicated the relative abundance, and shape of the plots indicated their affiliation. Venn diagrams showed the shared ASV number of archaea (A) and bacteria (B) in different sites.

It was clearly illustrated in the ternary plotting on species level that most archaea and bacteria species were plotted at the corners or edges, showing clear site-specific distribution pattern. Most of them were specific to one or two site, and only 11 archaea amplicon sequence variants (ASVs) and 17 bacteria ASVs were present at all three sites. Compared to 32 archaea ASVs and 85 bacteria ASVs specifically inhabited at AS site, 799 archaea ASVs and 477 bacteria ASVs at ES site, and 762 archaea ASVs and 145 bacteria ASVs at NS site. ES and NS sites shared 196 archaea ASVs and 94 bacteria ASVs, which is much more than they shared with the AS site (Fig. 3A, B). Ternary plotting clearly illustrated the archaea and bacteria distribution patterns on species level. For archaea community, 36 of 54 species were specific at ES and NS sites, comprising 52.8% of Euryarchaeota, 27.8% of Thaumarchaeota, and 19.4% of other archaea (Fig. 3A, Table S2). For bacteria community, 133 of 180 species were specific at ES and NS sites. Among them, 37.6%, 12.0%, 9.0%, and 7.5% were affiliated with Proteobacteria, Planctomycetes, Chloroflexi, and Firmicutes (Fig. 3B, Table S3).

### Correlation of prokaryotes and keystone species selection.

**FIG 4:**
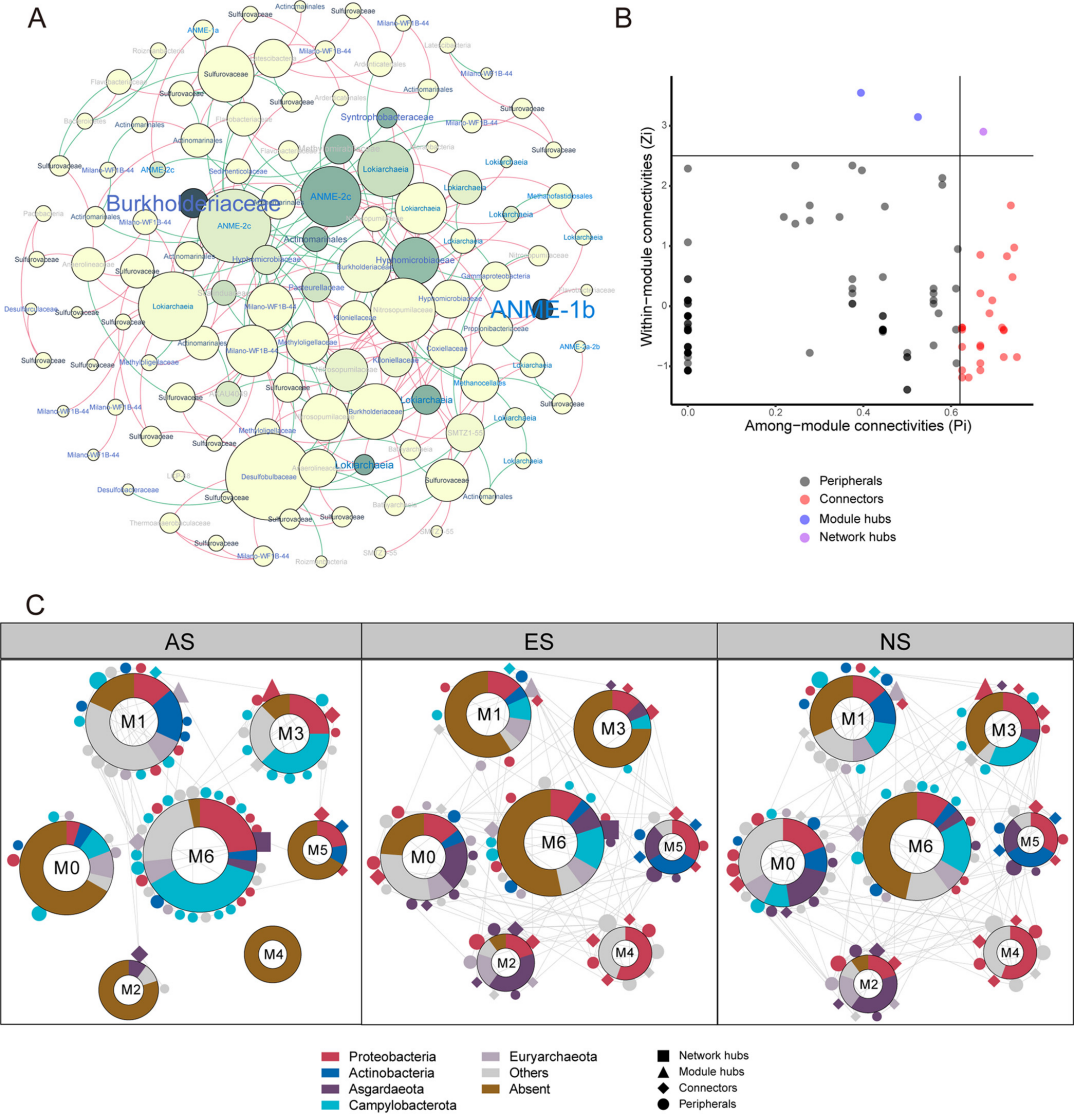
Correlation of archaea and bacteria species. (A) Molecular ecological network (MEN) constructed with archaea and bacteria ASVs of all samples (*n* = 18). The node size indicated the node degree. Color of the nodes indicated its relative abundance at ES site. Links between nodes suggested positive (red) and negative (green) correlations between nodes. The labels annotated the lineage of the nodes, whose color indicated their affiliation. (B) Identification of keystone species via the Zi-Pi analysis. (Network hubs, Zi ≥ 2.5, Pi ≥ 0.62; Module hubs, Zi ≥ 2.5, Pi < 0.62; Connectors, Zi < 2.5, Pi ≥ 0.62; Peripherals, Zi < 2.5, Pi < 0.62). (C) Seven modules were separated by the leading eigenvector of the community matrix, and they were specific to different sites. Color of the nodes indicated their affiliation, and shape of them represented their roles in network. Links between nodes suggested the correlation between the nodes at the present stage.

Interspecies correlation of archaea and bacteria was evaluated via molecular ecological network analysis (MENA) with samples from all stages (*n* = 18). A total of 117 nodes representing prokaryotic species and 257 links representing the node-between correlations were in the network, displaying scale-free, small-world, and modular properties (Table S3). We found that the ASVs with higher relative abundance (indicated by darker color) are less connective (indicated by node size). ANME-2c, Desulfobulbaceae, Milano-WF1B-44, Lokiarchaeia, and Nitrosopumilaceae were highly connective (Fig. 4A). One network hub, 2 module hub, and 24 connectors were identified, which were defined as keystone species (28, 29), and 24 of them were present at ES site (Fig. 4B, Table S4). Among the keystone species, 40.7%, 18.5%, and 14.8% affiliated with Proteobacteria, Asgardaeota, and Euryarchaeota. Seven modules (M) were separated by the leading eigenvector of community (30). The modules were dominantly comprised of Proteobacteria, Actinobacteria, Asgardaeota, Campylobacterota, and Euryarchaeota, but their compositions varied between different modules. However, these modules seemed to function in different sites. More than half of the species in M0, M2, M4, and M5 were present at ES site, and Proteobacteria and Asgardaeota were dominant species in these modules, suggesting these species may be functioning at this site. Species in M1, M3, and M6 were dominantly comprising of Proteobacteria and Campylobacterota, and they were prevalent at AS site. At NS site, all species in M0, M4, M5, and a majority number of species in other modules were present (Fig. 4C).

### Carbon, sulfur, and nitrogen cycling at different sites of Haima cold seep.

**FIG 5:**
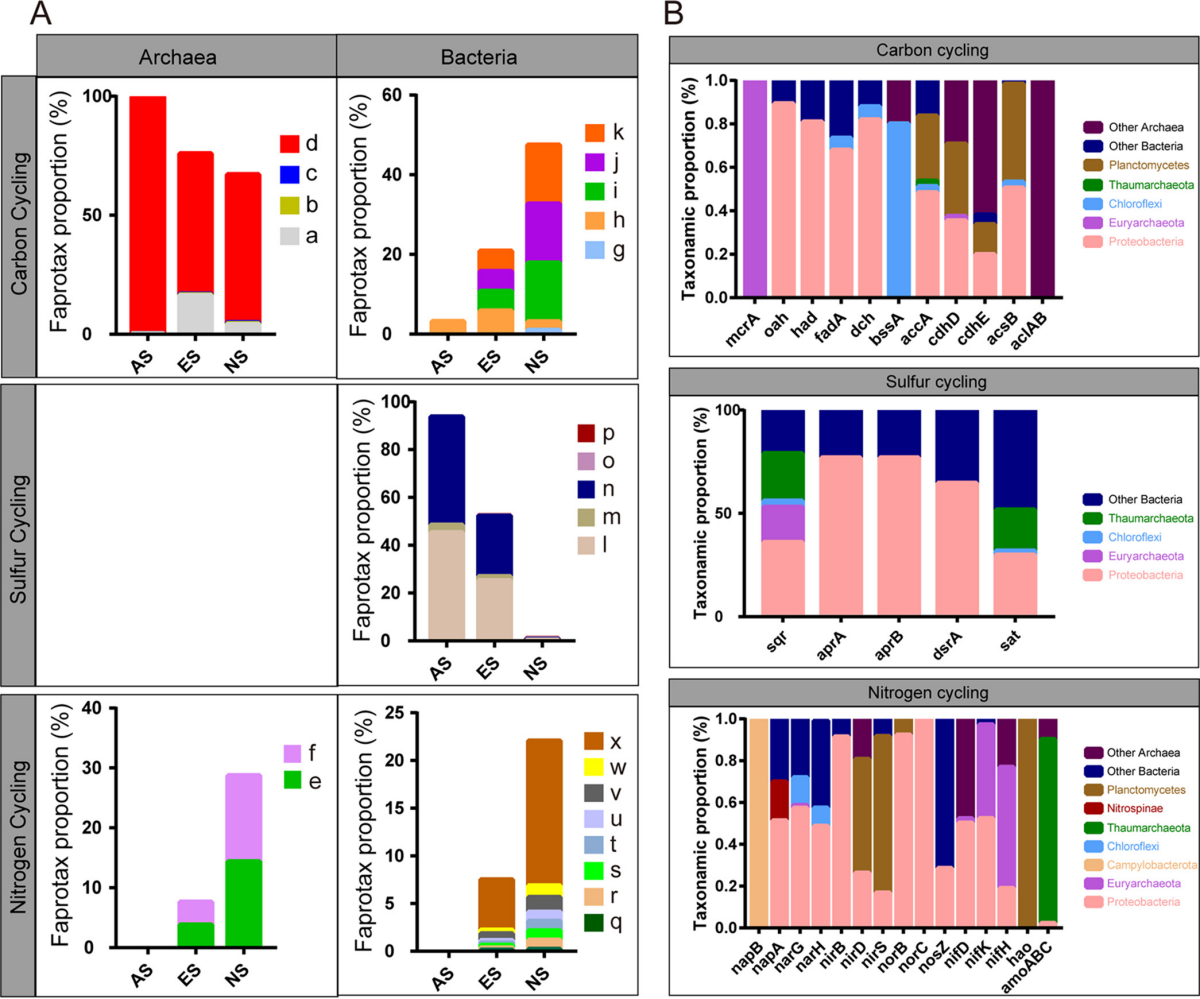
Carbon, sulfur, and nitrogen metabolisms. (A) Functional prediction for archaea and bacteria with Faprotax database. (B) Taxonomic affiliation of key genes involved in carbon, sulfur, and nitrogen metabolisms identified by metagenomic data. a. methanogenesis by disproportionation of methyl groups; b. methanogenesis by CO_2_ reduction with H_2_; c. hydrogenotrophic methanogenesis; d. methanogenesis; e. aerobic ammonia oxidation; f. nitrification; g. chitinolysis; h. fermentation; i. aromatic hydrocarbon degradation; j. aromatic compound degradation; k. hydrocarbon degradation; l. sulfate respiration; m. sulfite respiration; n. respiration of sulfur compounds; o. dark thiosulfate oxidation; p. dark oxidation of sulfur compounds; q. denitrification; r. nitrate ammonification; s. nitrite ammonification; t. nitrite respiration; u. nitrate respiration; v. nitrate reduction; w. nitrogen respiration; x. ureolysis.

To evaluate the differences in geochemical cycling between three sites, we predicted the functions of the archaea and bacteria with ASVs by Faprotax. Archaea were involved in carbon cycling (i.e., methanogenesis) and nitrogen cycling (i.e., aerobic ammonia oxidation and nitrification). The proportion of ASVs involved in carbon cycling showed decline at ES and NS sites, while obviously increased in nitrogen cycling. Bacteria participated in carbon cycling (i.e., chitinolysis, fermentation, aromatic hydrocarbon degradation, aromatic compound degradation, and hydrocarbon degradation), sulfur cycling (i.e., sulfate respiration, sulfite respiration, respiration of sulfur compounds, dark thiosulfate oxidation, and dark oxidation of sulfur compounds), and nitrogen cycling (i.e.,denitrification, nitrate ammonification, nitrite ammonification, nitrite respiration, nitrate respiration, nitrate reduction, nitrogen respiration, and ureolysis). The proportion of ASVs involved in carbon and nitrogen cycling showed obviously increased at ES site compared to AS site, while that of sulfur cycling declined (Fig. 5A).

As followed, we clarified the metabolic pathways for carbon, sulfur, and nitrogen cycling and taxonomic affiliation of their key genes at ES site via metagenomic approach. For carbon cycling, the reverse methanogenesis pathway for anaerobic oxidation of methane (AOM), and the benzol-CoA degradation pathway and β oxidation pathway for aromatic or chain hydrocarbon oxidation were identified. The key gene for AOM (*mcrA*) was all belonging to Euryarchaeota, while the benzoyl-CoA degradation pathway genes (*dch*, *oah*, *had*) and β-oxidation pathway gene (*fadA*) were dominantly affiliated with Proteobacteria. The *bssA* gene encoding benzylsuccinate synthase, initiating oxidation of aromatic hydrocarbons, were belonging to Chloroflexi and Archaea (Fig. 5B). Complete reductive tricarboxylic acid (rTCA) cycle and Wood-Ljungdahl (WL) pathway were probably responsible for carbon fixation at this site, implicating complete carbon cycling at ES site. Genes in WL pathway (*accA*, *cdhD*, *cdhE*, *acsB*) were dominantly belonging to Proteobacteria, Planctomycetes, and archaea, while the *aclA/B* genes for rTCA pathway were belonging to archaea (Fig. 5B). For sulfur cycling, the assimilatory and dissimilatory sulfate reduction (ASR and DSR) pathways were identified, whose genes were dominantly belonging to Proteobacteria (Fig. 5B). Sulfide oxidation via sulfide: quinone oxidoreductase was present since the presence of the *sqr* gene (Fig. 5B). However, the genes in SOX system responsible for sulfite and thiosulfate oxidation, and sulfide oxidation sulfide dehydrogenase encoded by gene *fccB* were absent, suggesting that sulfur cycle was incomplete at ES site. For nitrogen cycling, the pathways of dissimilatory nitrate reduction to ammonia (DNRA), denitrification, nitrogen fixation, and nitrification were identified, suggesting a complete nitrogen cycle at ES site. nitrate reduction genes (*napB*, *napA*, *narG*, *narH*) were carried by Campylobacterota, Proteobacteria, Nitrospinae, and Chloroflex. Further reduction via DNRA (*nirB* and *nirD*) and denitrification (*nirS*, *norB*, *norC*, and *nosZ*) was probably mediated by Proteobacteria and Planctomycetes. Nitrogen fixation genes (*nifD*, *nifH*, and *nifK*) were dominantly carried by Proteobacteria and Euryarchaeota. Nitrification genes (*hao*, *amoABC*) were dominantly affiliated with Planctomycetes and Thaumarchaeota (Fig. 5B).

## DISCUSSION

Cold seeps are well known for their amazing prosperous fauna communities in dark high-pressive deep-sea once thought to be a flat and lifeless desert. Less attentions have been paid on the declining extinct seeps. Like the whalefall as a gift for deep-sea colonizers, fluid nourishes the deep-sea organisms for even thousands of years after its extinction (9). In this study, we uncovered the unique composition and diversity of prokaryotes in an extinct seep site and revealed the role of them in biogeochemical cycling and sustainment of extinct seep ecosystem. Furthermore, many extinct seep specific species and networked keystone lineages were classified as Proteobacteria, suggesting its significance in extinct seeps.

Fauna communities in extinct seeps is significantly different from the active seeps. The species relying on their chemoautotrophic symbionts (i.e., clams and mussels) were replaced by the heterotrophic ones (i.e., sea anemones, cold-water corals, and sponges) (5). A dramatic decline in richness, diversity, as well as biomass of fauna community was found in extinct seeps (8). However, the changes in prokaryotic diversity were not in accordance with that of fauna, even opposite to it. We surprisingly found the increase of alpha diversity of archaea in extinct seep site, indicating the significance of geofluid for the archaea lineages here. Composition of archaea may change quickly after fluid ceased, since we observed the ES samples were either clustered with the AS ones or the NS ones, resulting in non-statistic difference between ES community with AS and NS (Fig. 2D, Table 1). Samples from locations with potential weak fluid maintained the archaea composition as active seeps, while the communities did not benefit from the fluid might quickly transited to be as the non-seep ones, suggesting the archaea community was typically fluid shaped (20). The methane fluid made strong natural selection for archaea community, which eliminates all but the best-adapted species, namely, the ANMEs in active seeps (31). Additionally, the activities of benthic animals, such as the movement of clams and ampharetid polychaetes, and the tube structure of tubeworm could create channels for geofluid and made significant modification to sediment environment (5, 32–34). Changes in environmental factors (i.e., methane, alkalinity, nutrient resources) that may limit the growth of prokaryotes from active to extinct sites probably resulted in emergence of new niches that can be occupied by the previous rare lineages (31, 35, 36). This was supported by the previous finding that the rare taxa Nitrososphaera in active seep site was alkalinity intolerant (37). On the other hand, diversity of bacteria did not change significantly during transition from active to extinct seep, but its composition varied between different sites, suggesting its potential functional flexibility during transition of prokaryotic community. More shared ASVs with ES and NS sites may suggest higher similar environment of these sites to some extent. We noticed that the highly connective nodes in network (i.e., ANME-2c, Desulfobulbaceae, Milano-WF1B-44, and Nitrosopumilaceae) were usually chemoautotrophs, suggesting their potential metabolic interactions with other species. Moreover, the specific species to ES site, keystone species were all dominated by Proteobacteria (Fig. 3 and 4), and they were probably functioning in all modules of the network. These clues indicated the significance of Proteobacteria in community structuring.

**FIG 6:**
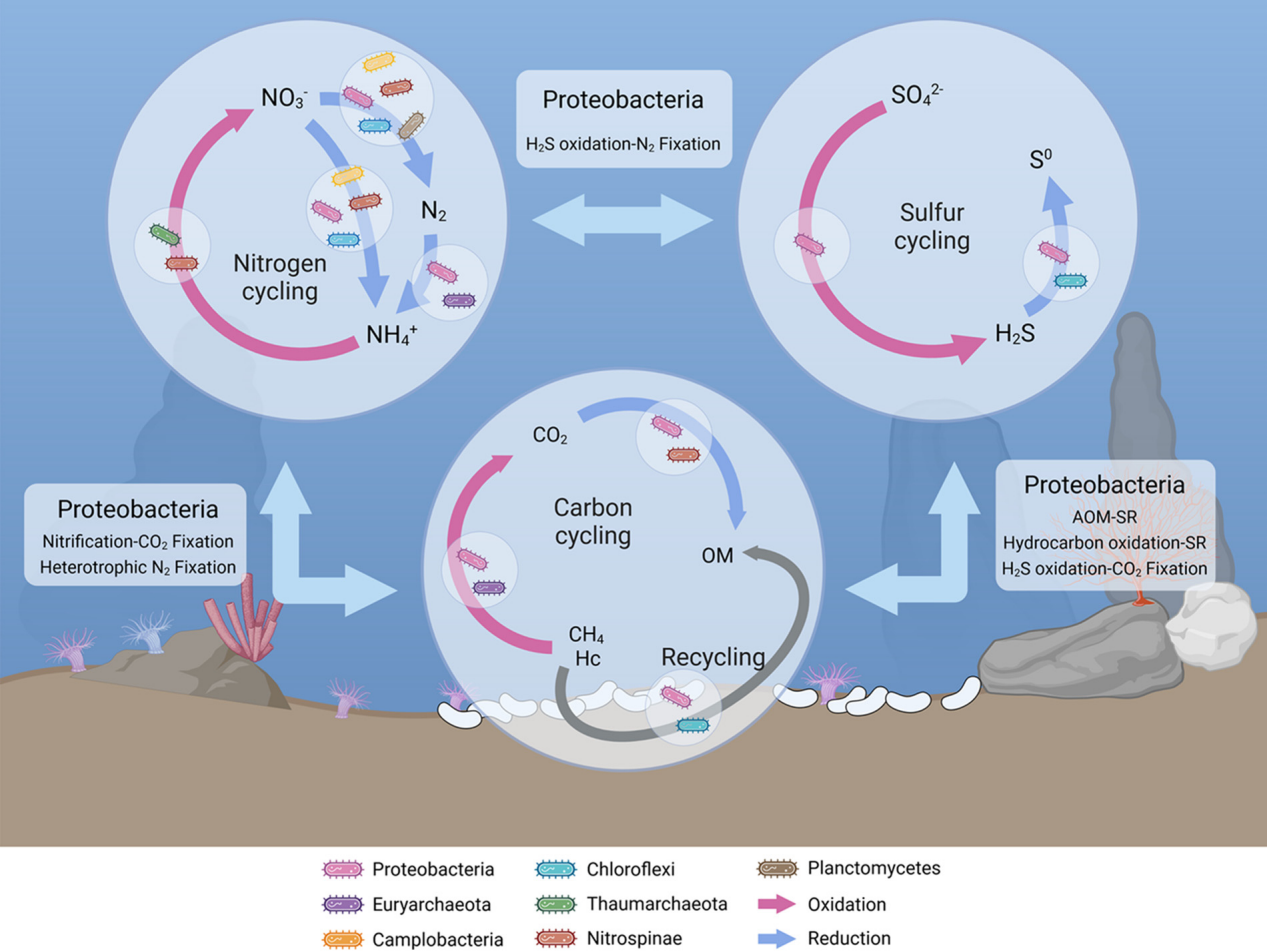
Proposed model for carbon, sulfur, and nitrogen cycling in extinct seep. Diverse lineages are getting involved in metabolisms related to geochemical cycling. Carbon and nitrogen cycling are supposed to be complete in extinct seeps, while sulfur cycle is incomplete, lacking the potential for sulfur oxidation to sulfate. Proteobacteria species show potential to couple carbon, sulfur, and nitrogen cycling at extinct seep, and therefore be proposed as keystones of the microbiome. Hc, hydrocarbon; OM, organic matter; AOM, anaerobic oxidation of methane; SR, sulfate reduction.

Variation in prokaryotic community composition caused pronounced changes in biogeochemical cycling in extinct seep, especially the emergence of nitrogen cycling. Combination of Faprotax functional prediction and metagenomic approaches revealed that carbon cycling and organic carbon recycling were both present in extinct seep site. Both approaches suggested that methane consumption via the classic reverse methanogenesis pathway was mediated by ANMEs (38). Carbonates derived from AOM latterly reacts with calcium and generates autogenetic carbonate rocks, constructing the representative seabed landscape of extinct seeps (12, 16–18, 39). Non-methane hydrocarbons, inclusive of aromatic and chain hydrocarbons, were potentially oxidated by Proteobacteria, Chloroflexi via the benzoyl-CoA and β-oxidation pathways (25, 40, 41). The abundant JS-1 clade affiliated with Atribacteria was also capable to ferments nonmethane hydrocarbons into organic acids (42). Previous researches have proved that rTCA and WL pathways are the most effective pathway for carbon fixation (43, 44), which were only identified by metagenome, carried by Proteobacteria, etc. at ES site, rather than the functional prediction approach. One thing we should notice that several Proteobacteria lineages were able to degrade hydrocarbons via a reverse WL pathway, indicating the WL pathway here could either for carbon fixation or hydrocarbon degradation (45, 46). As followed, we found sulfur cycling was not complete in extinct seep in this study with both approaches. The sulfate reduction pathways (ASR and DSR) were identified and unsurprisingly carried by Proteobacteria, which partially explained the longevity tubeworms depending on sulfide and more gradual decline of its population after ceasassion of seep fluid (5). However, the sulfur oxidation components were absent, except for sulfide:quinone oxidoreductase encoded by *sqr* gene, implying the conversion of hydrogen sulfide into polysulfide (47), rather than sulfur cycling. It was reported previously that Euryarchaeota and Thaumarchaeota were *sqr* carrying archaea (48), but little was known about their roles in environmental sulfur cycling, which was supported by our Faprotax functional prediction results (Fig. 5A). However, regarding that they are usually sulfide tolerant, *sqr* may help them with cellular sulfide detoxification (49). We are surprising to find that both approaches suggested the presence of complete nitrogen cycling in the extinct seep site, and the proportion of involving taxa kept increasing to the nonseep site (Fig. 5A). Proteobacteria was deeply getting involved in nitrogen cycling, including but not limited to DNRA, denitrification, and nitrification (Fig. 5B). The new emerging Nitrososphaera affiliated with Thaumarchaeota was an chemolithoautotrophic ammonia-oxidizing archaea carrying the *amoABC* genes (37), which was in accordance with our results of both approaches (Fig. 5B). Therefore, results of Faprotax functional prediction and metagenome were roughly consistent in our study, indicating they were both powerful approaches and complementary to each other. Most of dominant taxa were involved in biogeochemical cycling in extinct seep (Fig. 6). Increased prokaryotic diversity caused diversity of metabolic patterns, for example the emergence of nitrogen cycling.

Although the metabolic profiles of prokaryotes in extinct seep are quite different from the inactive sulfide chimney due to the discrepancy in fluid composition, we can still seek some inspiration of the microbiology ecological process from the inactive vents. The ‘mineral-shaped’ microbiome is sustained by the by-products of venting (20, 50, 51), reminding us of the hydrocarbons, the ‘by-products’ of thermal methanogenesis in cold seep fluid (52). Moreover, nutrients, such as ammonium, are also terming as by-products of methanogenesis by disproportion of organic matter (53). Key species at inactive vents, such as Gammaproteobacteria and Nitrospirae, are able to couple the geochemical cycles together, serving as producers. We are exiting to find that members in Proteobacteria are able to couple the cycles of carbon, sulfur, and nitrogen. Many Deltaproteobacterial SRB are partners for ANMEs, coupling AOM and SR indirectly (54). Other SRB members in hydrocarbon-rich environment, such as oil and hydrocarbon seeps, adopting diverse metabolic mechanisms for aromatics and alkane oxidation directly coupling to SR (46, 55–59). Gammaproteobacteria members are able to couple fixation of inorganic carbon to sulfur oxidation (60). Moreover, Proteobacteria are also ubiquitous nitrifiers that carrying the *cbbL* gene, coupling nitrification with carbon fixation via the CBB cycle (61, 62). Recently, proteobacterial nitrogen fixers, who are dominantly heterotrophs, are identified to be abundant in surface ocean (63), indicating the recycling of organic carbon compounds in support of nitrogen cycling. Lastly, sulfur compounds are usually promoters for nitrifying bacteria (64–66), suggesting the coupling of sulfur and nitrogen cycling. A group of Proteobacterial nitrogen fixers identified in the surface ocean are classified as SRB (63), and many of them are carrying the genes for both sulfide oxidation and DNRA, nitrification, or nitrogen fixation (59, 63), suggesting their potential to couple sulfur cycling and nitrogen fixation. Meanwhile, many works have demonstrated the active sulfide oxidation coupled nitrogen reduction in Proteobacteria members (65). Taken together, we proposed the function diverse Proteobacteria as the keystone taxa in extinct seep, coupling the geochemical cycles and sustaining the prokaryotic community with energy and resources.

Collectively, we have clarified the diversity, taxonomic specificity, interspecies correlation, and metabolic profiles of sediment prokaryotes in an extinct seep. Different to the faunal community, prokaryotic diversity exhibits increase (archaea) or unchanged (bacteria) in extinct seep. They can either live chemolithoautotrophically on cycling of inorganic carbon, sulfur, and especially nitrogen that is not existing in active seeps, or chemoorganotrophically. Accordingly, we proposed a model for prokaryotic metabolic profiles in extinct seep (Fig. 6). It is worth noting that many of the extinct seep specific and keystone lineages are classified as Proteobacteria. Regarding the functional diversity and metabolic flexibility of this clade, Proteobacteria may be an integrator of geochemical cycles and play critical role in energy and resource supplement in support with the stability of microbiome in extinct seep.

## MATERIALS AND METHODS

### Sample collection.

Sediment samples were obtained from Haima cold seep in the north of South China Sea (16.9° N, 110.4° E, Fig. 1) in September 2020. Pushcores were retrieved with the help of Haima remote operational vehicle (ROV), one (24 cm) from an active seeping site (AS, 1440 m), four (6 cm, 5 cm, 8 cm, 11 cm) from an extinct seepage site (ES, 1353 m), and one (17 cm) from a nonseep site (NS, 1393 m, Fig. 1). Pushcores longer than 10 cm were sliced into 3 cm per sample, and the ones shorter than 10 cm were used as a sample (Table S1). All samples were immediately frozen with liquid nitrogen and then stored at −80°C before use. The images were shot by the camara on the Haima ROV and the maps were illustrated with Ocean Data View (version 5.4.0).

### DNA extraction.

To avoid the vertical heterogeneity effect of prokaryotic communities, only the top six samples (0 to 18 cm) for the AS site pushcore were used. So, six samples for each site were used for DNA extraction. Total DNA was extracted from 2 g sediments using the TGuide S96 Magnetic Soil/Stool DNA kit (Tiangen Biotech [Beijing] Co., Ltd.) according to manufacturer’s instructions. The DNA concentration of the samples was measured with the Qubit dsDNA HS assay kit and Qubit 4.0 Fluorometer (Invitrogen, Thermo Fisher Scientific, OR, USA).

### Full-length 16S rRNA gene amplicon sequencing.

Bacterial and archaeal 16S rRNA genes were amplified using the 27F/1492R primer pairs (67), and 20F/1492R primer pairs (67, 68), respectively. Amplifications were conducted with the extracted DNA as the template, barcoded primer pairs, and KOD OneTM PCR Master Mix (Toyobo, Japan) in a 30 μL content. PCR progress was shown as followed: 95°C for 2 min; followed by 25 cycles of 98°C for 10 s, 55°C for 30 s, 72°C for 90 s; and finished with 72°C for 2 min. Amplicons were purified with Agencourt AMPure XP Beads (Beckman Coulter, Indianapolis, IN) and quantified using the Qubit dsDNA HS assay kit and Qubit 4.0 Fluorometer (Invitrogen, Thermo Fisher Scientific, OR, USA). After the individual quantification, amplicons were pooled in equal amounts for library construction. Libraries were prepared with SMRTbell Express Template Prep kit 2.0 (Pacific Biosciences) and sequenced on PacBio Sequel II platform.

### Prokaryotic diversity analysis.

Raw data were primarily filtered and demultiplexed using the SMRT Link software (version 8.0) with the minPasses ≥5 and minPredictedAccuracy ≥0.9, and the chimeras were removed by lima (v1.7.0) software to obtain the circular consensus sequencing (CCS) reads. Reads without primers and over length range (1,200 to 1,650 bp) were discarded and quality filtering using the Cutadapt (version 2.7) (69). Amplicon sequence variants (ASVs) were generated with DADA2 (70) in QIIME2 (71) and the ones with relative abundance <0.005% were filtered (72). The ASV matrices were rarefied by the minimum sample size for archaea (5714) and bacteria (7847). Taxonomy annotation was performed based on the Naive Bayes classifier in QIIME2 (71) using the SILVA database (73) (release 132) with a confidence threshold of 70%. Alpha diversity indices were calculated with Picante (74) package in R. Weighted unifrac dissimilarity based PCoA, ANOSIM, permutational multivariate analysis of variance were performed via vegan package (75) in R (with 999 permutations for ANOSIM and PERMANOVA). Weighted unifrac distance based UPGMA cluster analysis and ternary plotting were perform via BMKCloud (Biomarker Technologies Co., Ltd., Beijing, China).

### Functional prediction analysis.

Functional prediction of prokaryotic communities was conducted with the Faprotax, a manually constructed database mapping prokaryotic taxa to metabolic or other ecologically relevant functions based on the literature on cultured representatives (76). The prediction was obtained from the normalized archaeal and bacterial 16S rRNA ASV tables annotated against the SILVA v132 database.

### Molecular ecological network analysis.

To identify the interactions of prokaryotic species at different stages, molecular ecological networks (MENs) were constructed on the basis of Pearson correlations of bacteria and archaea ASVs abundances, followed by an RMT-based approach that determines the correlation cutoff threshold in an automatic fashion (77–79) with the Molecular Ecological Network Analyses Pipeline (MENAP) (80). Archaeal and bacterial ASVs from the same sample were combined for construction of MENs (*n* = 18), and only ASVs present in at least 6 samples were included for correlation calculation. The topological indices were calculated in the MENAP interface (80). Modules were separated following the leading eigenvector of the community matrix (30). Random networks were generated for each empirical network by randomly rewiring the links among the nodes while constraining n and L, following the Maslov–Sneppen procedure (81) in the MENAP. Within-module connectivity (Zi) and among-module connectivity (Pi) of each node were calculated with MENAP for classification of its topological roles. Module hubs (Zi ≥ 2.5, Pi < 0.62), connectors (Zi < 2.5, Pi ≥ 0.62), and network hubs (Zi ≥ 2.5, Pi ≥ 0.62) were identified and defined as keystone nodes (28, 29). Other nodes were defined as peripherals.

### Metagenomic analysis.

10 nanogram of DNA was used to produce library via VAHTS Universal Plus DNA Library Pren kit for Illumina and the pooled libraries were sequenced on Illumina Novaseq 6000 platform. A total of 7,103,829,330 bases of 18,195,418 raw reads was primarily filtered by Trimmomatic (version 0.33, PE LEADING:3 TRAILING:3 SLIDINGWINDOW:50:20 MINLEN:120) (82) to obtain clean reads and then assembled via MEGAHIT (83) (version v1.1.2). Contigs less than 300 bp were removed. Identification of opening reading frame (ORF) was conducted via MetaGeneMark (version 3.26). Nonredundant genes were clustered in line with sequence similarity of 95% and coverage of 90% by MMseq2 (version 11-e1a1c). Taxonomic and functional annotation were performed with Nr, Swiss-Prot and Kyoto Encyclopedia of Genes and Genomes (KEGG) databases by amino acid sequence blasting in DIAMOND (version 0.9.29). To construct the metabolic pathway, genes were mapped via Reconstruct tool in KEGG (84). The Schematic diagram was generated via BioRender (https://app.biorender.com) and modified manually.

### Data availability.

The amplicon sequencing data were deposited at the NCBI Sequence Read Archive under the BioProject PRJNA846870 for the AS samples (SAMN28906174-SAMN28906179 and SAMN28906134-SAMN28906139) and PRJNA847028 for ES and NS samples. The metagenome sequencing data were deposited at the NCBI Sequence Read Archive under the BioProject PRJNA847028.
