# Supplementary material for: Riddles of Lost City: Chemotrophic Prokaryotes Drives Carbon, Sulfur, and Nitrogen Cycling at an Extinct Cold Seep, South China Sea
Source: Microbiol Spectr. 2022 Dec 13;11(1):e03338-22. doi: 10.1128/spectrum.03338-22 (PMC9927161; doi:10.1128/spectrum.03338-22)
Supplement: Supplemental file 1 — Fig. S1, Movie S1 legend, and Tables S1 to S5. Download spectrum.03338-22-s0001.pdf, PDF file, 0.5 MB [file spectrum.03338-22-s0001.pdf]

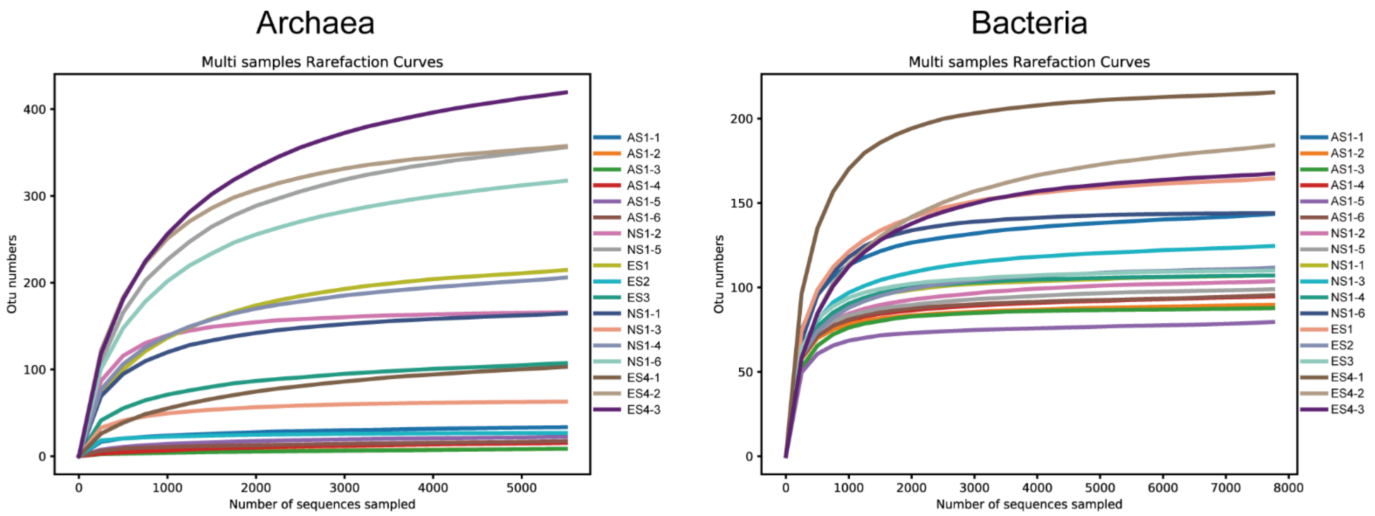

Supplementary Figure 1. Rarefaction curve of archaea and bacteria samples after subsample.

Supplementary movie. Seafloor landscape of the extinct site (ES) at Haima cold seep, SCS. Mussel and clam remains, carbonate rocks were found in this site, but live benthic animals were hardly seen. The video was shot by Haima remote operative vehicle (ROV) in September 2020.

Supplementary Table 1. Samples description

Pushcore	Sample	Depth (cm)	16S rRNA amplicon		Metagenome	Site
			Bacteria	Archaea		
NS1	NS1-1	0-3	√	√		NS
	NS1-2	3-6	√	√		NS
	NS1-3	6-9	√	√		NS
	NS1-4	9-12	√	√		NS
	NS1-5	12-15	√	√		NS
	NS1-6	15-18	√	√		NS
AS1	AS1-1	0-3	√	√		AS
	AS1-2	3-6	√	√		AS
	AS1-3	6-9	√	√		AS
	AS1-4	9-12	√	√		AS
	AS1-5	12-15	√	√		AS
	AS1-6	15-18	√	√		AS
ES1	ES1	0-6	√	√		ES
ES2	ES2	0-5	√	√	√	ES
ES3	ES3	0-8	√	√		ES
ES4	ES4-1	0-3	√	√		ES
	ES4-2	3-6	√	√		ES
	ES4-3	6-11	√	√		ES

Supplementary Table 2 Archaeal species relative abundance and distributional ratio in different sites

Phylum	Species	AS		ES		NS	
		RelAbu	Ratio	RelAbu	Ratio	RelAbu	Ratio
Euryarchaeota	Methanogenium_cariaci	5.83E-05	1.00	0.00E+00	0.00	0.00E+00	0.00
Euryarchaeota	uncultured_bacterium_g_ANME-2b	4.34E-01	0.99	3.21E-04	0.00	2.42E-03	0.01
Nanoarchaeaeota	uncultured_bacterium_c_Nanohaloarchaeia	8.75E-05	0.75	2.92E-05	0.25	0.00E+00	0.00
Euryarchaeota	uncultured_bacterium_f_ANME-1a	1.12E-02	0.73	2.57E-03	0.17	1.66E-03	0.11
Euryarchaeota	uncultured_bacterium_f_ANME-1b	4.69E-01	0.54	3.70E-01	0.42	3.44E-02	0.04
Euryarchaeota	Methanogenium_frigidum	4.38E-04	0.52	0.00E+00	0.00	4.08E-04	0.48
Euryarchaeota	uncultured_bacterium_f_ANME-2c	6.79E-02	0.48	4.93E-02	0.35	2.53E-02	0.18
Asgardaeota	uncultured_bacterium_c_Odinarchaeia	1.28E-03	0.06	9.86E-03	0.43	1.18E-02	0.51
Asgardaeota	uncultured_bacterium_p_Asgardaeota	4.08E-04	0.05	4.49E-03	0.60	2.57E-03	0.34
Euryarchaeota	uncultured_bacterium_o_Methanosarcinales	1.17E-04	0.05	1.46E-04	0.06	2.30E-03	0.90
Asgardaeota	uncultured_bacterium_c_Lokiarchaeia	1.39E-02	0.02	1.86E-01	0.29	4.49E-01	0.69
Euryarchaeota	uncultured_bacterium_g_ANME-3	1.20E-03	0.02	4.23E-02	0.74	1.34E-02	0.24
Nanoarchaeaeota	uncultured_bacterium_o_Deep_Sea_Euryarchaeotic_GroupDSEG	5.83E-05	0.01	2.45E-03	0.22	8.84E-03	0.78
uncultured_bacterium	uncultured_bacterium_k_Archaea	2.92E-05	0.00	3.47E-03	0.20	1.38E-02	0.80
Euryarchaeota	uncultured_bacterium_f_ANME-2a-2b	2.92E-05	0.00	1.38E-02	0.70	5.92E-03	0.30
Crenarchaeota	uncultured_bacterium_c_Bathyarchaeia	8.75E-05	0.00	6.66E-02	0.73	2.47E-02	0.27
Euryarchaeota	uncultured_bacterium_o_Marine_Benthic_Group_D_and_DHVEG-1	5.83E-05	0.00	4.38E-02	0.45	5.46E-02	0.55
Thaumarchaeota	uncultured_bacterium_g_Candidatus_Nitrosopumilus	0.00E+00	0.00	1.17E-04	1.00	0.00E+00	0.00
Thaumarchaeota	Candidatus_Nitrosopumilus_piranensis	0.00E+00	0.00	2.92E-05	0.02	1.63E-03	0.98

Thaumarchaeota	uncultured_bacterium_c_Marine_Benthic_Group_A	0.00E+00	0.00	1.05E-03	0.45	1.28E-03	0.55
Thaumarchaeota	Candidatus_Nitrosopumilus_koreensis	0.00E+00	0.00	8.75E-05	0.27	2.33E-04	0.73
Thaumarchaeota	Candidatus_Nitrosopumilus_adriaticus	0.00E+00	0.00	6.50E-03	0.16	3.31E-02	0.84
Thaumarchaeota	uncultured_bacterium_f_Nitrososphaeraceae	0.00E+00	0.00	8.75E-05	1.00	0.00E+00	0.00
Thaumarchaeota	uncultured_bacterium_f_Nitrosopumilaceae	0.00E+00	0.00	2.16E-02	0.19	9.19E-02	0.81
Thaumarchaeota	Candidatus_Nitrosopumilus_salaria	0.00E+00	0.00	7.58E-04	0.26	2.16E-03	0.74
Thaumarchaeota	uncultured_bacterium_c_Nitrososphaeria	0.00E+00	0.00	0.00E+00	0.00	3.21E-04	1.00
Thaumarchaeota	uncultured_bacterium_g_Candidatus_Nitrosopelagicus	0.00E+00	0.00	0.00E+00	0.00	2.04E-04	1.00
Nanoarchaeaeota	uncultured_bacterium_o_Aenigmarchaeales	0.00E+00	0.00	1.46E-04	0.03	4.46E-03	0.97
Hydrothermarchaeota	uncultured_bacterium_p_Hydrothermarchaeota	0.00E+00	0.00	1.37E-03	0.14	8.34E-03	0.86
Hadesarchaeaeota	uncultured_bacterium_p_Hadesarchaeaeota	0.00E+00	0.00	9.33E-04	0.10	8.81E-03	0.90
Euryarchaeota	uncultured_bacterium_o_Marine_Group_II	0.00E+00	0.00	5.83E-05	0.02	3.68E-03	0.98
Euryarchaeota	uncultured_bacterium_f_Rice_Cluster_II	0.00E+00	0.00	4.96E-04	1.00	0.00E+00	0.00
Euryarchaeota	uncultured_bacterium_o_Methanocellales	0.00E+00	0.00	1.13E-02	0.12	8.15E-02	0.88
Euryarchaeota	Methanocella_arvoryzae	0.00E+00	0.00	2.92E-05	1.00	0.00E+00	0.00
Euryarchaeota	uncultured_bacterium_o_SG8-5	0.00E+00	0.00	6.71E-04	0.09	6.91E-03	0.91
Euryarchaeota	Methanococcoides_vulcani	0.00E+00	0.00	1.17E-04	1.00	0.00E+00	0.00
Euryarchaeota	Methanococcoides_alaskense	0.00E+00	0.00	4.17E-03	0.76	1.34E-03	0.24
Euryarchaeota	uncultured_bacterium_o_Methanomassiliicoccales	0.00E+00	0.00	5.83E-05	1.00	0.00E+00	0.00
Euryarchaeota	uncultured_bacterium_o_Methanofastidiosales	0.00E+00	0.00	5.05E-03	0.43	6.62E-03	0.57
Euryarchaeota	uncultured_bacterium_o_ANME-1	0.00E+00	0.00	1.75E-04	0.75	5.83E-05	0.25
Euryarchaeota	uncultured_bacterium_o_Marine_Group_III	0.00E+00	0.00	0.00E+00	0.00	2.04E-04	1.00
Euryarchaeota	uncultured_bacterium_g_Methanimicrococcus	0.00E+00	0.00	1.27E-01	1.00	0.00E+00	0.00

Euryarchaeota	uncultured_bacterium_f_Methermicoccaceae	0.00E+00	0.00	3.50E-04	0.60	2.33E-04	0.40
Euryarchaeota	uncultured_bacterium_f_Marine_group_II_euryarchaeote_REDSEA-S19_B7N8	0.00E+00	0.00	0.00E+00	0.00	3.50E-04	1.00
Euryarchaeota	uncultured_bacterium_c_Thermoplasmata	0.00E+00	0.00	8.90E-03	0.41	1.27E-02	0.59
Euryarchaeota	uncultured_bacterium_o_Methanomicrobiales	0.00E+00	0.00	2.92E-05	1.00	0.00E+00	0.00
Euryarchaeota	uncultured_bacterium_f_Methanoperedenaceae	0.00E+00	0.00	1.49E-03	0.66	7.58E-04	0.34
Euryarchaeota	uncultured_bacterium_p_Euryarchaeota	0.00E+00	0.00	0.00E+00	0.00	1.46E-04	1.00
Euryarchaeota	uncultured_bacterium_g_Methanocella	0.00E+00	0.00	0.00E+00	0.00	1.75E-04	1.00
Crenarchaeota	uncultured_bacterium_f_Geothermarchaeaceae	0.00E+00	0.00	9.92E-04	0.64	5.54E-04	0.36
Crenarchaeota	uncultured_bacterium_o_miscellaneous_Crenarchaeota_group_archaeon_SMTZ1-55	0.00E+00	0.00	1.04E-02	0.11	8.10E-02	0.89
Asgardaeota	uncultured_bacterium_o_Candidatus_Heimdallarchaeota_archaeon_LC_3	0.00E+00	0.00	0.00E+00	0.00	2.92E-05	1.00
Asgardaeota	uncultured_bacterium_c_Heimdallarchaeia	0.00E+00	0.00	2.33E-04	0.50	2.33E-04	0.50

Supplementary Table 3 Bacterial species relative abundance and distributional ratio in different sites

Phylum	Species	AS		ES		NS	
		RelAbu	Ratio	RelAbu	Ratio	RelAbu	Ratio
Acetothermia	uncultured_bacterium_c_Acetothermiia	0.00E+00	0.00	6.37E-04	1.00	0.00E+00	0.00
Acidobacteria	uncultured_bacterium_c_ODP1230B23.02	0.00E+00	0.00	1.49E-04	1.00	0.00E+00	0.00
Acidobacteria	uncultured_bacterium_o_Aminicenantales	2.12E-04	0.00	4.29E-02	0.84	8.16E-03	0.16
Acidobacteria	uncultured_bacterium_g_Subgroup_23	8.24E-03	0.53	6.86E-03	0.44	3.19E-04	0.02
Acidobacteria	uncultured_bacterium_c_Subgroup_9	0.00E+00	0.00	0.00E+00	0.00	2.71E-02	1.00
Acidobacteria	uncultured_bacterium_g_Subgroup_10	0.00E+00	0.00	0.00E+00	0.00	2.12E-02	1.00
Actinobacteria	uncultured_bacterium_c_RBG-16-55-12	0.00E+00	0.00	6.37E-05	1.00	0.00E+00	0.00
Actinobacteria	uncultured_bacterium_c_WCHB1-81	6.37E-05	0.00	2.82E-02	0.88	3.76E-03	0.12
Actinobacteria	Cutibacterium_namnetense	0.00E+00	0.00	8.50E-05	0.36	1.49E-04	0.64
Actinobacteria	Cutibacterium_acnes	0.00E+00	0.00	3.61E-04	0.13	2.34E-03	0.87
Actinobacteria	Gardnerella_vaginalis	0.00E+00	0.00	4.25E-05	0.12	3.19E-04	0.88
Actinobacteria	uncultured_bacterium_o_Actinomarinales	8.93E-02	0.33	5.08E-03	0.02	1.80E-01	0.66
Actinobacteria	Pseudarthrobacter_oxydans	0.00E+00	0.00	0.00E+00	0.00	4.25E-05	1.00
Aegiribacteria	uncultured_bacterium_p_Aegiribacteria	4.25E-04	0.38	4.25E-04	0.38	2.76E-04	0.25
Aerophobetes	uncultured_bacterium_c_Aerophobetes_bacterium_ SCGC_AAA255-F10	0.00E+00	0.00	2.93E-03	0.90	3.40E-04	0.10
Aerophobetes	uncultured_bacterium_p_Aerophobetes	0.00E+00	0.00	3.38E-03	0.86	5.31E-04	0.14
Atribacteria	uncultured_bacterium_c_JS1	1.10E-02	0.03	3.10E-01	0.94	1.04E-02	0.03
Bacteroidetes	uncultured_bacterium_f_Lentimicrobiaceae	0.00E+00	0.00	7.73E-03	1.00	0.00E+00	0.00
Bacteroidetes	uncultured_bacterium_o_SJA-28	0.00E+00	0.00	8.50E-05	1.00	0.00E+00	0.00
Bacteroidetes	Bacteroides_acidifaciens	0.00E+00	0.00	4.25E-04	1.00	0.00E+00	0.00
Bacteroidetes	Candidatus_Azobacteroides_pseudotrichonymphae	0.00E+00	0.00	1.34E-02	1.00	0.00E+00	0.00

Bacteroidetes	Bacteroides_dorei	0.00E+00	0.00	8.50E-05	1.00	0.00E+00	0.00
Bacteroidetes	uncultured_bacterium_o_Bacteroidales	2.12E-05	0.02	8.92E-04	0.98	0.00E+00	0.00
Bacteroidetes	Lutimonas_sp	1.49E-04	0.14	9.35E-04	0.86	0.00E+00	0.00
Bacteroidetes	Cloacibacterium_normanense	0.00E+00	0.00	5.52E-03	0.73	2.08E-03	0.27
Bacteroidetes	uncultured_bacterium_f_Bacteroidetes_BD2-2	2.15E-03	0.34	4.08E-03	0.64	1.27E-04	0.02
Bacteroidetes	uncultured_bacterium_g_Luteivirga	0.00E+00	0.00	3.82E-04	0.45	4.67E-04	0.55
Bacteroidetes	Lutimonas_saemankumensis	1.06E-03	0.62	6.58E-04	0.38	0.00E+00	0.00
Bacteroidetes	uncultured_bacterium_f_Muribaculaceae	0.00E+00	0.00	8.50E-05	0.25	2.55E-04	0.75
Bacteroidetes	uncultured_bacterium_g_Lutimonas	1.08E-02	0.76	3.33E-03	0.24	0.00E+00	0.00
Bacteroidetes	uncultured_bacterium_f_SB-5	1.91E-04	1.00	0.00E+00	0.00	0.00E+00	0.00
Bacteroidetes	uncultured_bacterium_f_Marinilabiliaceae	2.12E-05	1.00	0.00E+00	0.00	0.00E+00	0.00
BHI80-139	uncultured_bacterium_p_BHI80-139	0.00E+00	0.00	4.46E-04	0.45	5.52E-04	0.55
Chloroflexi	uncultured_bacterium_o_MSBL5	0.00E+00	0.00	8.28E-04	1.00	0.00E+00	0.00
Chloroflexi	uncultured_bacterium_o_Sh765B-AG-111	0.00E+00	0.00	9.35E-04	1.00	0.00E+00	0.00
Chloroflexi	uncultured_bacterium_o_vadinBA26	0.00E+00	0.00	2.55E-04	1.00	0.00E+00	0.00
Chloroflexi	uncultured_bacterium_f_AB-539-J10	0.00E+00	0.00	3.19E-04	1.00	0.00E+00	0.00
Chloroflexi	uncultured_bacterium_o_GIF9	0.00E+00	0.00	6.37E-05	1.00	0.00E+00	0.00
Chloroflexi	uncultured_bacterium_o_FW22	0.00E+00	0.00	1.15E-03	1.00	0.00E+00	0.00
Chloroflexi	uncultured_bacterium_o_DscP2	0.00E+00	0.00	3.63E-03	1.00	0.00E+00	0.00
Chloroflexi	uncultured_bacterium_o_SBR1031	4.25E-05	0.15	2.34E-04	0.85	0.00E+00	0.00
Chloroflexi	uncultured_bacterium_g_Thermoflexus	0.00E+00	0.00	9.77E-04	0.82	2.12E-04	0.18
Chloroflexi	uncultured_bacterium_g_SCGC-AB-539-J10	0.00E+00	0.00	4.67E-04	0.81	1.06E-04	0.19
Chloroflexi	uncultured_bacterium_c_Anaerolineae	0.00E+00	0.00	3.42E-03	0.71	1.42E-03	0.29
Chloroflexi	uncultured_bacterium_o_MSB-5B2	0.00E+00	0.00	7.20E-03	0.55	5.95E-03	0.45
Chloroflexi	uncultured_bacterium_o_Ardenticatenales	9.03E-03	0.82	1.89E-03	0.17	1.06E-04	0.01
Chloroflexi	uncultured_bacterium_f_Anaerolineaceae	6.39E-03	0.08	1.20E-02	0.15	6.37E-02	0.78

Chloroflexi	uncultured_bacterium_g_Pelolinea	1.32E-03	0.87	1.91E-04	0.13	0.00E+00	0.00
Chloroflexi	uncultured_bacterium_o_SJA-15	1.49E-04	1.00	0.00E+00	0.00	0.00E+00	0.00
Chloroflexi	uncultured_bacterium_o_S085	0.00E+00	0.00	0.00E+00	0.00	1.20E-02	1.00
CK-2C2-2	uncultured_bacterium_p_CK-2C2-2	0.00E+00	0.00	2.55E-04	1.00	0.00E+00	0.00
Cyanobacteria	uncultured_bacterium_o_Chloroplast	0.00E+00	0.00	8.50E-05	0.36	1.49E-04	0.64
Cyanobacteria	Brassica_napus_rape	0.00E+00	0.00	1.91E-04	0.24	5.95E-04	0.76
Cyanobacteria	Oryza_sativa_Japonica_Group_Japanese_rice	0.00E+00	0.00	8.50E-05	0.18	3.82E-04	0.82
Cyanobacteria	Medicago_truncatula_barrel_medic	0.00E+00	0.00	0.00E+00	0.00	1.15E-03	1.00
Dadabacteria	uncultured_bacterium_o_Dadabacteriales	0.00E+00	0.00	0.00E+00	0.00	1.04E-02	1.00
Dependentiae	uncultured_bacterium_f_UBA12409	0.00E+00	0.00	4.25E-05	1.00	0.00E+00	0.00
Dependentiae	uncultured_bacterium_f_Vermiphilaceae	0.00E+00	0.00	8.07E-04	0.08	8.84E-03	0.92
Elusimicrobia	uncultured_bacterium_c_4-29	0.00E+00	0.00	7.22E-04	1.00	0.00E+00	0.00
Camplobacteria	uncultured_bacterium_g_Arcobacter	0.00E+00	0.00	1.27E-04	1.00	0.00E+00	0.00
Camplobacteria	uncultured_bacterium_g_Sulfurovum	3.82E-01	0.61	2.41E-01	0.39	2.95E-03	0.00
Camplobacteria	Folliculinopsis_sp	8.05E-03	0.94	3.61E-04	0.04	1.70E-04	0.02
Firmicutes	uncultured_bacterium_f_Family_XIII	0.00E+00	0.00	2.55E-04	1.00	0.00E+00	0.00
Firmicutes	Lactobacillus_plantarum	0.00E+00	0.00	4.67E-04	1.00	0.00E+00	0.00
Firmicutes	Enterococcus_casseliflavus	0.00E+00	0.00	8.50E-05	1.00	0.00E+00	0.00
Firmicutes	Weissella_cibaria	0.00E+00	0.00	7.86E-04	1.00	0.00E+00	0.00
Firmicutes	uncultured_bacterium_f_Christensenellaceae	0.00E+00	0.00	2.12E-05	1.00	0.00E+00	0.00
Firmicutes	Lactobacillus_johnsonii	0.00E+00	0.00	8.92E-04	1.00	0.00E+00	0.00
Firmicutes	uncultured_bacterium_g_Enterococcus	0.00E+00	0.00	6.37E-05	1.00	0.00E+00	0.00
Firmicutes	uncultured_bacterium_f_GoM-GC232-4463-Bac1	6.37E-05	0.50	6.37E-05	0.50	0.00E+00	0.00
Firmicutes	Lactobacillus_buchneri	0.00E+00	0.00	0.00E+00	0.00	2.76E-04	1.00
Firmicutes	Lactobacillus_iners	0.00E+00	0.00	0.00E+00	0.00	4.67E-04	1.00
Firmicutes	Bacillus_aeolius	0.00E+00	0.00	0.00E+00	0.00	7.77E-02	1.00

Gemmatimonadetes	uncultured_bacterium_o_saltmarsh_clone_LCP-68	2.57E-03	0.88	3.61E-04	0.12	0.00E+00	0.00
Gemmatimonadetes	uncultured_bacterium_c_AKAU4049	0.00E+00	0.00	0.00E+00	0.00	9.26E-03	1.00
Gemmatimonadetes	uncultured_bacterium_c_PAUC43f_marine_benthic_group	0.00E+00	0.00	0.00E+00	0.00	5.62E-02	1.00
Kiritimatiellaeota	uncultured_bacterium_o_WCHB1-41	2.12E-05	0.50	2.12E-05	0.50	0.00E+00	0.00
Latescibacteria	uncultured_bacterium_f_Latescibacteraceae	0.00E+00	0.00	2.12E-04	0.77	6.37E-05	0.23
Latescibacteria	uncultured_bacterium_p_Latescibacteria	8.28E-04	0.29	2.00E-03	0.71	0.00E+00	0.00
Marinimicrobia_SAR406_clade	uncultured_bacterium_p_Marinimicrobia_SAR406_clade	0.00E+00	0.00	1.49E-04	1.00	0.00E+00	0.00
Omnitrophicaeota	uncultured_bacterium_p_Omnitrophicaeota	0.00E+00	0.00	1.25E-03	1.00	0.00E+00	0.00
Omnitrophicaeota	uncultured_bacterium_c_Omnitrophia	0.00E+00	0.00	2.12E-04	1.00	0.00E+00	0.00
Patescibacteria	uncultured_bacterium_p_Patescibacteria	0.00E+00	0.00	2.12E-05	1.00	0.00E+00	0.00
Patescibacteria	uncultured_bacterium_o_Candidatus_Falkowbacteria	0.00E+00	0.00	1.60E-02	1.00	0.00E+00	0.00
Patescibacteria	uncultured_bacterium_o_Candidatus_Collierbacteria	0.00E+00	0.00	4.25E-05	1.00	0.00E+00	0.00
Patescibacteria	uncultured_bacterium_c_Parcubacteria	0.00E+00	0.00	1.27E-04	1.00	0.00E+00	0.00
Patescibacteria	uncultured_bacterium_c_Gracilibacteria	0.00E+00	0.00	9.56E-04	1.00	0.00E+00	0.00
Patescibacteria	uncultured_bacterium_c_Microgenomatia	2.55E-04	0.11	1.98E-03	0.89	0.00E+00	0.00
Patescibacteria	uncultured_bacterium_o_Candidatus_Peregrinibacteria	2.12E-05	0.14	1.27E-04	0.86	0.00E+00	0.00
Patescibacteria	uncultured_bacterium_o_Candidatus_Woesebacteria	0.00E+00	0.00	1.51E-02	0.76	4.78E-03	0.24
Patescibacteria	uncultured_bacterium_c_WWE3	0.00E+00	0.00	2.40E-03	0.45	2.95E-03	0.55
Patescibacteria	uncultured_bacterium_o_Candidatus_Moranbacteria	1.82E-02	0.83	3.70E-03	0.17	0.00E+00	0.00

Patescibacteria	uncultured_bacterium_c_Kazania	2.12E-04	0.83	4.25E-05	0.17	0.00E+00	0.00
Patescibacteria	uncultured_bacterium_o_candidate_division_Kazan_bacterium_RBG_13_50_9	0.00E+00	0.00	4.25E-05	0.11	3.61E-04	0.89
Patescibacteria	uncultured_bacterium_o_Candidatus_Beckwithbacteria	0.00E+00	0.00	5.31E-04	0.08	5.93E-03	0.92
Patescibacteria	uncultured_bacterium_o_Candidatus_Roizmanbacteria	5.97E-03	1.00	0.00E+00	0.00	0.00E+00	0.00
Patescibacteria	uncultured_bacterium_c_Berkelbacteria	3.82E-04	1.00	0.00E+00	0.00	0.00E+00	0.00
Patescibacteria	uncultured_bacterium_o_Candidatus_Pacebacteria	3.55E-03	0.58	0.00E+00	0.00	2.59E-03	0.42
Patescibacteria	uncultured_bacterium_o_Candidatus_Chisholmbacteria	0.00E+00	0.00	0.00E+00	0.00	2.29E-02	1.00
Patescibacteria	uncultured_bacterium_o_Candidatus_Curtissbacteria	0.00E+00	0.00	0.00E+00	0.00	1.16E-02	1.00
Planctomycetes	uncultured_bacterium_f_SG8-4	0.00E+00	0.00	2.75E-02	1.00	0.00E+00	0.00
Planctomycetes	uncultured_bacterium_c_SPG12-343-353-B69	0.00E+00	0.00	6.80E-04	1.00	0.00E+00	0.00
Planctomycetes	uncultured_bacterium_o_CCM11a	0.00E+00	0.00	6.58E-04	1.00	0.00E+00	0.00
Planctomycetes	uncultured_bacterium_f_AKAU3564_sediment_group	4.25E-05	0.02	1.76E-03	0.98	0.00E+00	0.00
Planctomycetes	uncultured_bacterium_f_GWA2-50-13	0.00E+00	0.00	6.80E-04	0.43	8.92E-04	0.57
Planctomycetes	uncultured_bacterium_g_Candidatus_Scalindua	0.00E+00	0.00	3.46E-03	0.12	2.45E-02	0.88
Planctomycetes	uncultured_bacterium_f_Pirellulaceae	8.71E-04	0.28	1.70E-04	0.06	2.04E-03	0.66
Proteobacteria	Photobacterium_damselae	0.00E+00	0.00	1.51E-03	1.00	0.00E+00	0.00
Proteobacteria	uncultured_bacterium_f_Desulfobulbaceae	0.00E+00	0.00	1.30E-03	1.00	0.00E+00	0.00
Proteobacteria	Wolbachia_endosymbiont	0.00E+00	0.00	5.95E-04	1.00	0.00E+00	0.00
Proteobacteria	uncultured_bacterium_o_B2M28	0.00E+00	0.00	6.16E-04	1.00	0.00E+00	0.00
Proteobacteria	Bosea_robiniae	0.00E+00	0.00	2.12E-05	1.00	0.00E+00	0.00

Proteobacteria	uncultured_bacterium_g_Thiogranum	0.00E+00	0.00	1.40E-03	1.00	0.00E+00	0.00
Proteobacteria	Halomonas_pantelleriensis	0.00E+00	0.00	8.50E-05	1.00	0.00E+00	0.00
Proteobacteria	uncultured_bacterium_o_Sva0485	0.00E+00	0.00	1.04E-03	1.00	0.00E+00	0.00
Proteobacteria	uncultured_bacterium_f_Rhodobacteraceae	0.00E+00	0.00	6.37E-05	1.00	0.00E+00	0.00
Proteobacteria	uncultured_bacterium_f_Syntrophaceae	0.00E+00	0.00	1.49E-04	1.00	0.00E+00	0.00
Proteobacteria	Acinetobacter_haemolyticus	0.00E+00	0.00	2.12E-05	1.00	0.00E+00	0.00
Proteobacteria	Acinetobacter_calcoaceticus	0.00E+00	0.00	1.06E-04	1.00	0.00E+00	0.00
Proteobacteria	uncultured_bacterium_c_Deltaproteobacteria	0.00E+00	0.00	8.50E-04	1.00	0.00E+00	0.00
Proteobacteria	uncultured_bacterium_f_Clade_III	0.00E+00	0.00	3.40E-04	1.00	0.00E+00	0.00
Proteobacteria	uncultured_bacterium_g_Desulfoconvexum	0.00E+00	0.00	1.42E-03	1.00	0.00E+00	0.00
Proteobacteria	Donghicola_eburneus	0.00E+00	0.00	2.12E-04	1.00	0.00E+00	0.00
Proteobacteria	Escherichia_coli	0.00E+00	0.00	1.06E-04	1.00	0.00E+00	0.00
Proteobacteria	Sphingomonas_pseudosanguinis	0.00E+00	0.00	6.37E-05	1.00	0.00E+00	0.00
Proteobacteria	uncultured_bacterium_f_Thiotrichaceae	4.25E-05	0.04	9.13E-04	0.96	0.00E+00	0.00
Proteobacteria	uncultured_bacterium_g_SEEP-SRB2	0.00E+00	0.00	2.32E-03	0.95	1.27E-04	0.05
Proteobacteria	uncultured_bacterium_g_SEEP-SRB1	2.89E-03	0.04	6.94E-02	0.94	1.36E-03	0.02
Proteobacteria	uncultured_bacterium_g_Commensalibacter	0.00E+00	0.00	5.31E-04	0.89	6.37E-05	0.11
Proteobacteria	uncultured_bacterium_g_Desulfocapsa	1.02E-03	0.16	5.39E-03	0.84	0.00E+00	0.00
Proteobacteria	uncultured_bacterium_g_Desulfatiglans	3.48E-03	0.15	1.86E-02	0.82	4.67E-04	0.02
Proteobacteria	uncultured_bacterium_g_Desulfobulbus	1.78E-03	0.30	4.16E-03	0.70	0.00E+00	0.00
Proteobacteria	Aquabacterium_fontiphilum	0.00E+00	0.00	3.82E-04	0.67	1.91E-04	0.33
Proteobacteria	Hydrogenophaga_flava	0.00E+00	0.00	1.53E-03	0.64	8.71E-04	0.36
Proteobacteria	Tepidimonas_fonticaldi	0.00E+00	0.00	5.31E-04	0.52	4.89E-04	0.48
Proteobacteria	Ralstonia_insidiosa	0.00E+00	0.00	2.10E-03	0.48	2.29E-03	0.52
Proteobacteria	uncultured_bacterium_g_SEEP-SRB4	5.78E-03	0.58	4.12E-03	0.42	0.00E+00	0.00

Proteobacteria	uncultured_bacterium_o_Gammaproteobacteria_Incertae_Sedis	2.76E-03	0.57	1.95E-03	0.40	1.27E-04	0.03
Proteobacteria	uncultured_bacterium_c_Alphaproteobacteria	0.00E+00	0.00	2.76E-04	0.37	4.67E-04	0.63
Proteobacteria	Pseudomonas_fluorescens	0.00E+00	0.00	7.43E-04	0.26	2.12E-03	0.74
Proteobacteria	Halomonas_desiderata	0.00E+00	0.00	3.19E-04	0.25	9.77E-04	0.75
Proteobacteria	Brevundimonas_vesicularis	0.00E+00	0.00	1.06E-04	0.22	3.82E-04	0.78
Proteobacteria	Ralstonia_pickettii	0.00E+00	0.00	3.75E-02	0.21	1.45E-01	0.79
Proteobacteria	Acinetobacter_pittii	0.00E+00	0.00	4.25E-05	0.20	1.70E-04	0.80
Proteobacteria	Delftia_acidovorans	0.00E+00	0.00	4.25E-05	0.18	1.91E-04	0.82
Proteobacteria	Methyloceanibacter_methanicus	0.00E+00	0.00	2.12E-05	0.17	1.06E-04	0.83
Proteobacteria	Rodentibacter_ratti	0.00E+00	0.00	2.44E-03	0.15	1.38E-02	0.85
Proteobacteria	uncultured_bacterium_c_Gammaproteobacteria	0.00E+00	0.00	1.27E-04	0.15	7.43E-04	0.85
Proteobacteria	uncultured_bacterium_f_Methyloligellaceae	0.00E+00	0.00	2.08E-03	0.14	1.24E-02	0.86
Proteobacteria	Methyloceanibacter_marginalis	0.00E+00	0.00	4.67E-04	0.13	3.21E-03	0.87
Proteobacteria	uncultured_bacterium_o_NKB15	5.52E-04	0.90	6.37E-05	0.10	0.00E+00	0.00
Proteobacteria	uncultured_bacterium_f_Sedimenticolaceae	3.95E-03	0.88	3.40E-04	0.08	1.91E-04	0.04
Proteobacteria	uncultured_bacterium_f_Hyphomicrobiaceae	0.00E+00	0.00	3.33E-03	0.07	4.61E-02	0.93
Proteobacteria	Methyloceanibacter_superfactus	0.00E+00	0.00	1.06E-04	0.06	1.72E-03	0.94
Proteobacteria	uncultured_bacterium_f_Kiloniellaceae	0.00E+00	0.00	1.42E-03	0.06	2.42E-02	0.94
Proteobacteria	uncultured_bacterium_g_Coxiella	0.00E+00	0.00	3.19E-04	0.05	6.07E-03	0.95
Proteobacteria	uncultured_bacterium_f_Syntrophobacteraceae	0.00E+00	0.00	1.74E-03	0.04	4.28E-02	0.96
Proteobacteria	uncultured_bacterium_o_AT-s2-59	0.00E+00	0.00	2.34E-04	0.03	8.37E-03	0.97
Proteobacteria	uncultured_bacterium_o_Milano-WF1B-44	4.10E-01	0.97	9.26E-03	0.02	5.10E-03	0.01
Proteobacteria	uncultured_bacterium_f_Desulfobacteraceae	2.78E-03	0.96	0.00E+00	0.00	1.06E-04	0.04
Proteobacteria	uncultured_bacterium_o_UBA10353_marine_group	0.00E+00	0.00	0.00E+00	0.00	1.08E-03	1.00

Proteobacteria	Methylobacterium_brachiatum	0.00E+00	0.00	0.00E+00	0.00	1.70E-04	1.00
Proteobacteria	uncultured_bacterium_g_HIMB11	0.00E+00	0.00	0.00E+00	0.00	1.06E-04	1.00
Proteobacteria	uncultured_bacterium_g_Woeseia	0.00E+00	0.00	0.00E+00	0.00	1.24E-02	1.00
Proteobacteria	Serratia_marcescens	0.00E+00	0.00	0.00E+00	0.00	3.55E-03	1.00
Proteobacteria	uncultured_bacterium_o_MBMPE27	0.00E+00	0.00	0.00E+00	0.00	3.67E-03	1.00
Proteobacteria	uncultured_bacterium_g_Sva0081_sediment_group	0.00E+00	0.00	0.00E+00	0.00	5.73E-04	1.00
Proteobacteria	Aliidiomarina_maris	0.00E+00	0.00	0.00E+00	0.00	6.37E-05	1.00
Rokubacteria	uncultured_bacterium_g_wb1-A12	0.00E+00	0.00	1.49E-04	0.00	6.73E-02	1.00
Spirochaetes	uncultured_bacterium_g_Sediminispirochaeta	0.00E+00	0.00	5.52E-04	1.00	0.00E+00	0.00
Spirochaetes	uncultured_bacterium_f_Spirochaetaceae	0.00E+00	0.00	6.16E-04	0.88	8.50E-05	0.12
Spirochaetes	uncultured_bacterium_g_Spirochaeta_2	0.00E+00	0.00	8.71E-04	0.49	9.13E-04	0.51
TA06	uncultured_bacterium_p_TA06	6.58E-04	0.05	1.07E-02	0.87	9.98E-04	0.08
Tenericutes	uncultured_bacterium_g_Candidatus_Bacilloplasma	0.00E+00	0.00	7.65E-04	1.00	0.00E+00	0.00
Tenericutes	uncultured_bacterium_f_Mycoplasmataceae	0.00E+00	0.00	1.55E-03	1.00	0.00E+00	0.00
uncultured_bacterium	uncultured_bacterium_k_Bacteria	0.00E+00	0.00	3.61E-04	1.00	0.00E+00	0.00
Verrucomicrobia	Akkermansia_muciniphila	0.00E+00	0.00	1.27E-03	0.66	6.58E-04	0.34
WS1	uncultured_bacterium_p_WS1	8.50E-05	1.00	0.00E+00	0.00	0.00E+00	0.00
WS2	uncultured_bacterium_p_WS2	1.06E-04	1.00	0.00E+00	0.00	0.00E+00	0.00
Zixibacteria	uncultured_bacterium_p_Zixibacteria	0.00E+00	0.00	8.71E-04	1.00	0.00E+00	0.00

Supplementary Table 4 Topological indices for the molecular ecological network (MEN)

**Network Indexes**	**Empirical Network**	**100 Random Networks**
Total nodes	117	
Total links	257	
Cutoff	0.92	
R square of power-law	0.814	
Average degree (avgK)	4.393	
Average clustering coefficient (avgCC)	0.211	0.057 +/- 0.016
Average path distance (GD)	3.675	3.262 +/- 0.057
Geodesic efficiency (E)	0.325	0.350 +/- 0.004
Harmonic geodesic distance (HD)	3.081	2.856 +/- 0.031
Centralization of degree (CD)	0.119	0.119 +/- 0.000
Centralization of betweenness (CB)	0.175	0.147 +/- 0.019
Centralization of stress centrality (CS)	0.44	0.426 +/- 0.056
Centralization of eigenvector centrality (CE)	0.251	0.241 +/- 0.029
Transitivity (Trans)	0.16	0.063 +/- 0.011
Connectedness (Con)	1	0.992 +/- 0.018
Efficiency	0.97	0.970 +/- 0.001
Modularity	0.468	0.347 +/- 0.029

Supplementary Table 5 Keystone ASVs identified by network analysis

Role	ASVs	Zi	Pi	Kingdom	Phylum	Class	Order
N	ARC79	2.90	0.67	Archaea	Asgardaeota	Lokiarchaeia	Unclassified
M	ARC7	3.15	0.52	Archaea	Euryarchaeota	Methanomicrobia	Methanosarcinales
M	BAC823	3.58	0.53	Bacteria	Proteobacteria	Deltaproteobacteria	Desulfobacterales
C	BAC1560	0.21	0.67	Bacteria	Actinobacteria	Acidimicrobiia	Actinomarinales
C	BAC168	-1.07	0.67	Bacteria	Actinobacteria	Acidimicrobiia	Actinomarinales
C	ARC32	-0.68	0.67	Archaea	Asgardaeota	Lokiarchaeia	Unclassified
C	ARC9	-0.39	0.72	Archaea	Asgardaeota	Lokiarchaeia	Unclassified
C	ARC125	0.83	0.73	Archaea	Asgardaeota	Lokiarchaeia	Unclassified
C	ARC170	-0.36	0.63	Archaea	Asgardaeota	Lokiarchaeia	Unclassified
C	BAC319	0.10	0.69	Bacteria	Chloroflexi	Anaerolineae	Anaerolineales
C	ARC1139	-0.95	0.67	Archaea	Crenarchaeota	Bathyarchaeia	Unclassified
C	BAC250	-0.85	0.72	Bacteria	Camplobacteria	Campylobacteria	Campylobacterales
C	ARC8	1.68	0.74	Archaea	Euryarchaeota	Methanomicrobia	Methanosarcinales
C	ARC226	-0.36	0.63	Archaea	Euryarchaeota	Thermococci	Methanofastidiosales
C	BAC867	-0.85	0.75	Bacteria	Gemmatimonadetes	AKAU4049	Unclassified
C	BAC82	-1.19	0.63	Bacteria	Planctomycetes	Brocadiae	Brocadiales
C	BAC1	-0.40	0.72	Bacteria	Proteobacteria	Gammaproteobacteria	Betaproteobacteriales
C	BAC26	0.85	0.67	Bacteria	Proteobacteria	Gammaproteobacteria	Milano-WF1B-44
C	BAC682	-1.19	0.64	Bacteria	Proteobacteria	Gammaproteobacteria	Pasteurellales
C	BAC64	-0.36	0.72	Bacteria	Proteobacteria	Alphaproteobacteria	Rhizobiales
C	BAC172	-0.65	0.67	Bacteria	Proteobacteria	Alphaproteobacteria	Rhodovibrionales
C	BAC1177	0.97	0.74	Bacteria	Proteobacteria	Gammaproteobacteria	Betaproteobacteriales
C	BAC1289	-1.07	0.63	Bacteria	Proteobacteria	Gammaproteobacteria	Chromatiales
C	BAC182	-0.12	0.69	Bacteria	Proteobacteria	Alphaproteobacteria	Rhizobiales

C	BAC1757	-0.68	0.63	Bacteria	Proteobacteria	Alphaproteobacteria	Rhizobiales
C	BAC205	0.48	0.74	Bacteria	Proteobacteria	Gammaproteobacteria	Milano-WF1B-44
C	ARC810	-0.39	0.63	Archaea	Thaumarchaeota	Nitrososphaeria	Nitrosopumilales

N, network hub; M, module hub; C, connectors; Zi, within−module connectivities; Pi, among−module connectivities.
